# The light chain of tetanus toxin bound to arginine-rich cell-penetrating peptide inhibits cortical reaction in mouse oocytes

**DOI:** 10.3389/fcell.2023.1259421

**Published:** 2023-11-15

**Authors:** Omar G. Klinsky, Paula A. Wetten, Emilia Zanni-Ruiz, Martín A. Pavarotti, María Victoria Berberian, Marcela A. Michaut

**Affiliations:** ^1^ Laboratorio de Biología Reproductiva y Molecular, Instituto de Histología y Embriología de Mendoza (IHEM), Universidad Nacional de Cuyo-Consejo Nacional de Investigaciones Científicas y Técnicas (CONICET), Mendoza, Argentina; ^2^ Facultad de Ciencias Médicas, Universidad Nacional de Cuyo, Mendoza, Argentina; ^3^ Laboratorio de Transporte Intracelular, Instituto de Histología and Embriología de Mendoza (IHEM), Universidad Nacional de Cuyo-Consejo Nacional de Investigaciones Científicas y Técnicas (CONICET), Mendoza, Argentina; ^4^ Facultad de Ciencias Exactas y Naturales, Universidad Nacional de Cuyo, Mendoza, Argentina; ^5^ Instituto Interdisciplinario de Ciencias Básicas, Facultad de Ciencias Exactas y Naturales, Universidad Nacional de Cuyo, Consejo Nacional de Investigaciones Científicas y Técnicas (CONICET), Mendoza, Argentina

**Keywords:** cortical granules, tetanus toxin, arginine-rich cell-penetrating peptides, exocytosis, oocytes, cortical reaction, biotechnology

## Abstract

**Introduction:** Cortical reaction is a secretory process that occurs after a spermatozoon fuses with the oocyte, avoiding the fusion of additional sperm. During this exocytic event, the cortical granule membrane fuses with the oocyte plasma membrane. We have identified several molecular components involved in this process and confirmed that SNARE proteins regulate membrane fusion during cortical reaction in mouse oocytes. In those studies, we microinjected different nonpermeable reagents to demonstrate the participation of a specific protein in the cortical reaction. However, the microinjection technique has several limitations. In this work, we aimed to assess the potential of cell-penetrating peptides (CPP) as biotechnological tools for delivering molecules into oocytes, and to evaluate the functionality of the permeable tetanus toxin (bound to CPP sequence) during cortical reaction.

**Methods:** Arginine-rich cell-penetrating peptides have demonstrated the optimal internalization of small molecules in mammalian cells. Two arginine-rich CPP were used in the present study. One, labeled with 5-carboxyfluorescein, to characterize the factors that can modulate its internalization, and the other, the permeable light chain of tetanus toxin, that cleaves the SNAREs VAMP1 and VAMP3 expressed in mouse oocytes.

**Results:** Results showed that fluorescent CPP was internalized into the oocyte cytoplasm and that internalization was dependent on the concentration, time, temperature, and maturation stage of the oocyte. Using our functional assay to study cortical reaction, the light chain of tetanus toxin bound to arginine-rich cell-penetrating peptide inhibited cortical granules exocytosis.

**Discussion:** Results obtained from the use of permeable peptides demonstrate that this CPP is a promising biotechnological tool to study functional macromolecules in mouse oocytes.

## 1 Introduction

In mammals, the cortical reaction is a secretory process that occurs after a spermatozoon fuses with the oocyte during fertilization. This fusion event activates various molecular mechanisms in oocytes that are globally called oocyte activation. The first and more rapid sign of oocyte activation is the liberation of millions of zinc atoms, known as zinc sparks (Kim et al., 2011). After zinc sparks and in the following 15 min, granules localized in the oocyte cortical region -cortical granules-fuse with the oocyte plasma membrane (Cappa et al., 2018). This process is an exocytotic event triggered by an increase in cytoplasmatic calcium levels and avoids the fusion of additional spermatozoa. Therefore, cortical granules exocytosis -also named cortical reaction-is essential for ensuring the development of the preimplantation embryo.

Membrane fusion during cortical granule exocytosis is regulated by SNARE proteins. Our laboratory has made significant contributions to the identification of several proteins involved in this exocytosis process. We have demonstrated the participation of alpha-SNAP and NSF (Paola et al., 2015), Rab3A (Bello et al., 2016), as well as VAMP1 and VAMP3 (Paola et al., 2021). Furthermore, other groups have identified SNAP23 (Mehlmann et al., 2019), Rabphilin 3A (Masumoto et al., 1996), Rab27A (Wang et al., 2016), and Synaptotagmin I (Zhu et al., 2020). Collectively, these findings have allowed us to propose a comprehensive working model for membrane fusion during cortical granule exocytosis in mouse oocytes (Paola et al., 2021).

To identify the participation of a specific protein in the cortical reaction, we developed a functional assay based on cortical granules quantification during the cortical reaction parthenogenetically activated in mouse oocytes. To inhibit protein function, we performed microinjections of recombinant proteins, blocking antibodies, or toxins prior to parthenogenetic activation with strontium chloride (Paola et al., 2015; Bello et al., 2016; Paola et al., 2021). So far, oocyte microinjection is the standard method for introducing molecules into oocytes. However, this technique is associated with several limitations such as invasiveness, time-consuming nature, the requirement of specialized microinjection equipment, and skilled personnel (Rubino et al., 2016).

An alternative approach involves the use of cell-penetrating peptides (CPP), which are short peptides that have the capability to permeate cell membranes and deliver molecules directly into the cytoplasm. CPPs are derived from naturally occurring proteins and can be synthesized using solid-phase peptide synthesis. They can be used to deliver a wide range of cargo molecules, including proteins, nucleic acids, and small molecules, into the cell cytoplasm (Copolovici et al., 2014; Jafari et al., 2015; Dinca et al., 2016; Zhang et al., 2016; Mayorga et al., 2020; Xie et al., 2020).

One advantage of CPPs is their ease of use. Peptides can be readily synthesized and delivered into the cell cytoplasm using simple methods such as incubating cells in a CPP containing solution. In addition, this approach is less invasive than microinjection, and does not require specialized equipment or skilled personnel. Therefore, this investigation aims to ascertain whether CPP-mediated delivery can be utilized as a substitute method to intracytoplasmic microinjection for achieving intracellular delivery of macromolecules into oocytes.

Arginine-rich cell-penetrating peptides have demonstrated the optimal internalization of small molecules in mammalian cells (Mitchell et al., 2000; Mueller et al., 2008; Tünnemann et al., 2008). To assess the potential of cell-penetrating peptides as biotechnological tools for molecules deliver into oocytes, we first analyzed the ability of arginine-rich cell-penetrating peptide to cross both the zona pellucida and plasma membrane in immature and mature mouse oocytes, and then, determined the functionality of the light chain of tetanus toxin bound to this cell-penetrating peptide in the cortical granule exocytosis assay.

## 2 Materials and methods

### 2.1 Ethics statement

All animals were cared in accordance with the Guiding Principles in the Care and Use of Animals of the US National Institute of Health. All procedures were approved by the Institutional Animal Care and Use Committee of the School of Medical Science, Universidad Nacional de Cuyo (Protocol N° 169/2019).

### 2.2 Chemical and reagents

All chemicals and reagents, unless stated otherwise, were purchased from Sigma-Aldrich Chemical Inc. (St. Louis, United States).

### 2.3 Oocyte collection

Germinal Vesicle (GV, immature) and Metaphase II (MII, mature) oocytes were obtained from hormonally stimulated CF-1 female mice (8–12 weeks) and bred under controlled conditions of light and temperature. To obtain GV oocytes, females were injected intraperitoneally (i.p.) with 10 IU of purified equine chorionic gonadotropin hormone (PMSG) (Syntex, Argentina), and after 45–48 h cumulus oocyte-cell complexes (COC) were obtained by ovarian puncture. Earle’s balanced salt solution with 0.01% PVA, 0.001% Gentamicin, and 25 mM HEPES buffer, pH 7.3 (MEM/HEPES) was used as the collection medium, supplemented with 2.5 μM Milrinone to inhibit oocyte maturation. To denude the GV oocytes, cumulus cells were mechanically removed using fine-bore pipettes. MII oocytes were obtained from female mice stimulated with 10 IU (i.p.) of PMSG (Syntex, Argentina), followed by 10 IU (i.p.) of human chorionic gonadotropin hCG (Syntex, Argentina) 48 h later. After 13–17 h, the MII oocytes were obtained from the oviductal ampullae, denuded from cumulus cells by brief incubation in hyaluronidase (0.04%) and placed in CZB medium (81.62 mM NaCl, 4.83 mM KCl, 1.70 mM CaCl_2_.2H_2_O, 1.18 mM MgSO_4_.7H_2_O, 1.18 mM KH_2_PO_4_, 0.11 mM EDTA.2Na, 25.12 mM NaHCO_3_, 52 mM sodium lactate, 0.27 mM sodium pyruvate, 7 mM taurine, 1 mM L-glutamine, 10 mg/mL gentamicin, supplemented with 0.3% BSA) and covered with mineral oil in a humidified chamber (37°C, 5% CO_2_) for the shortest time possible until their use.

### 2.4 Cell penetrating peptide

The CPP used in this study possesses the following sequence: NH_2_-K-(5-FAM)RRRRRRRRRC-CONH_2_ (MW 2012,32 Da). It was custom-designed by our research team and was commercially synthesized by Innovagen AB using solid-phase synthesis, achieving a purity grade exceeding 98%. This CPP includes an amide group at the C-terminus of the cysteine to prevent undesired binding, a lysine residue at the N-terminus to provide a primary amino group suitable for protein conjugation, and a polyR sequence that confers a membrane-penetrating capability. Furthermore, 5-FAM(5-carboxyfluorescein) is conjugated covalently to CPP through the lysine residue, allowing the peptide to be visualized using fluorescence microscopy.

### 2.5 CPP incubations

**FIGURE 1:**
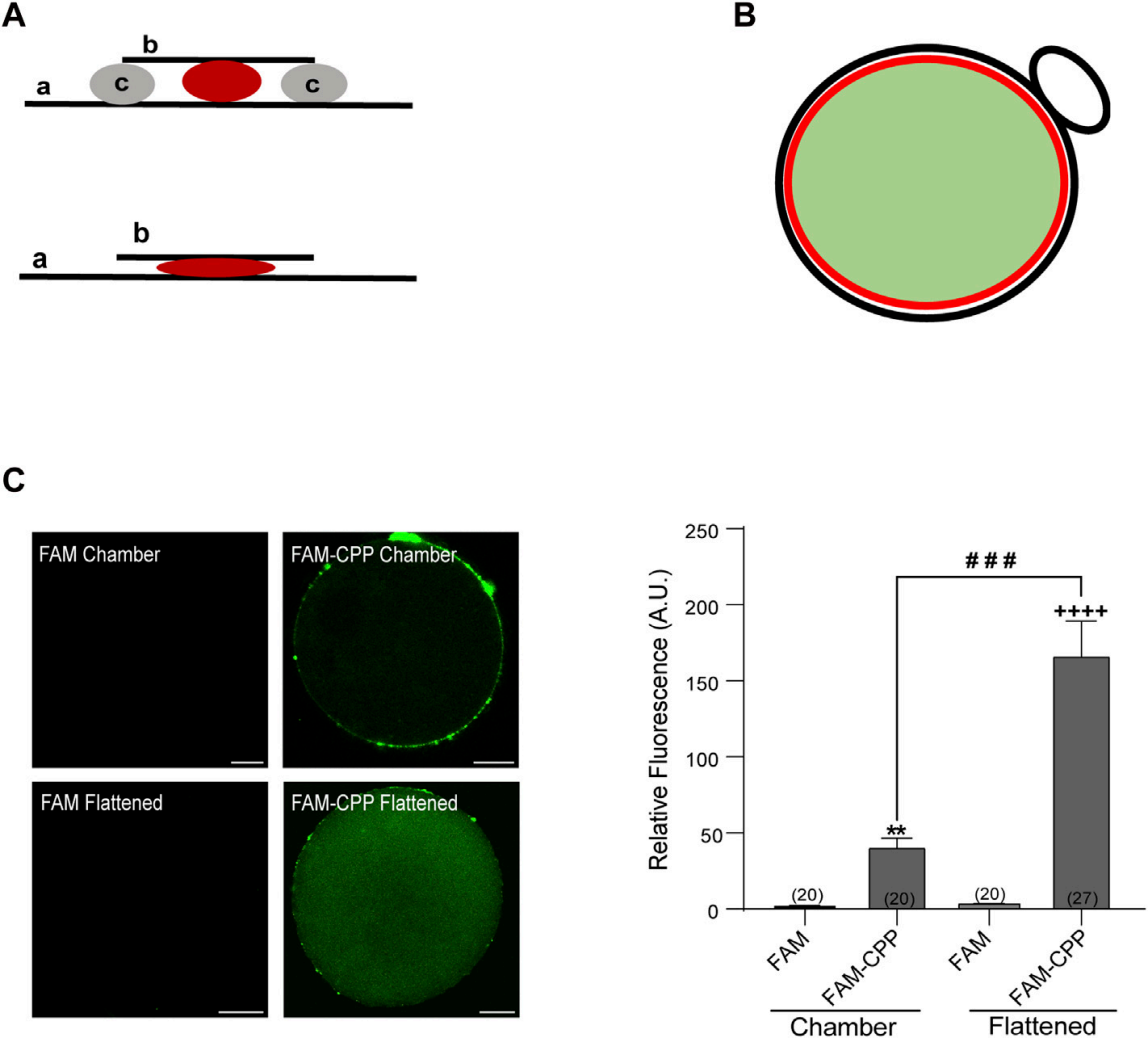
FAM-CPP is able to cross both zona pellucida and plasma membrane of mouse oocytes. **(A)** Schematic representation of mounting. Upper: chambered mounting. Lower: flattened mounting. **(A)** slide, **(B)** coverslip, **(C)** solid vaseline. **(B)** Schematic representation of absolute fluorescence quantification through Image J. Red circle represents the area taken to quantify the fluorescence intensity. It only takes the central area of the oocyte (light green area) and excludes the cortical area and polar body (black line). **(C)** Effect of mounting on the visualization of FAM-CPP internalization. MII oocytes with ZP were incubated with 10 μM FAM (control) or FAM-CPP during 1 h, washed, fixed, and mounted as indicated. Left: representative confocal microscopic images of oocytes taken at equatorial plane of the cell. Scale bar: 15 μm. Right: histogram showing relative fluorescence intensity (A.U.) for different mounting. The data represent mean ± SEM. Numbers in parentheses at the base of the bar represent the total number of oocytes. Comparisons between FAM and mounting types were made by Kruskal–Wallis and Dunn’s multiple comparisons test (*p* < 0.001). * (chamber) and + (flattened) represent significant differences compared to FAM. # represent significant differences between mounting types.

After collection, GV and MII oocytes were incubated in CPP in CZB medium. To check the effect of the zona pellucida (ZP) in the CPP internalization, some GV and MII oocytes were subjected to a brief incubation in acid Tyrode to remove the ZP, prior to carrying out the corresponding treatments with CPP. If not, oocytes were incubated with ZP at different concentrations of FAM-CPP: 1, 2 and 3 µM (only for MII oocytes), and 4, 10 and 16 µM (for GV and MII oocytes); at different times: 15, 30, and 60 min; and different temperatures: 37°C in a humidified atmosphere of CO_2_ (5%) or 4°C in a refrigerator. Likewise, as a control, a group of GV and MII oocytes was treated with CZB medium without CPP. Following, the ZP was removed by brief incubation in acid Tyrode (pH 2.2) and the oocytes were fixed in paraformaldehyde (3.7%) in Dulbecco’s PBS (DPBS) for 1 h at room temperature (RT). Cells were then washed in blocking solution (BS) containing 3 mg/mL BSA, 100 mM glycine, and 0.01% Tween 20 in DPBS, and mounted in Vectashield mounting medium (Vector Laboratories, Burlingame, CA) following two types of mounting techniques: in chamber or flattened (Figure 1A). Chamber mounting consists of placing two lines of solid vaseline between the slide and the coverslip to create a space where the oocytes are contained with a minimal compression to keep their spherical shape. On the contrary, in the flattened mounting, the lines of solid vaseline are absent and the cells are compressed between the slide and coverslip. Then, samples were sealed and stored at 4°C until viewing.

### 2.6 Endocytosis inhibition assays

To inhibit endocytosis, oocytes were incubated in CZB medium containing either 80 µM Dyngo-4a (Abcam) or 20 µM Cytochalasin B (Abcam) for 90 min at 37°C in a humidified atmosphere of 5% CO_2_. After washing, oocytes were incubated during 30 min in 10 µM FAM-CPP in the same conditions. Then, ZP was removed, and oocytes were fixed as described in section 2.5. Cells were separated in two groups. One cells group was flatten mounted to analyze the cytoplasmic FAM-CPP fluorescence. The other cells group was permeabilized with Triton X-100 for 15 min, stained with Rhodamine-Phalloidin 555 (1:1000) for 60 min and mounted in chamber. For DNA detection, Vectashield Mounting Medium (Vector Laboratories, Burlingame, CA) containing 1.5 μg/mL Hoechst 33342 (Molecular Probes, Invitrogen) was used. After sealing, slides were stored at 4°C until visualization.

### 2.7 Mitochondrial membrane potential (MMP) assay

MII oocytes with ZP were incubated with 10 µM FAM-CPP in CZB for 60 min, at 37 °C in a humidified atmosphere of CO_2_ (5%). Likewise, as a control, a group of MII oocytes was treated with 5-FAM (without CPP) in CZB. Next, MII oocytes were incubated for 30 min in a solution of 2 µM Tetramethylrhodamine perchlorate ethyl ester (TMRE), which allowed the staining of the mitochondrial membranes to determine their potential. Then, cells were washed twice in MEM/HEPES drops and transferred to a real-time chamber with MEM/HEPES without PVP to fix them on the glass and prevent their movement. *In vivo* visualization was performed on a laser scanning confocal microscope (FV1000, Olympus) using a UPLSAPO 20X/NA:0.75 objective lens, a pixel resolution of ×512512, and the pinhole fully open (800) to obtain the total fluorescence of the cells. Equatorial images of oocytes were captured using the same microscope setup. The absolute total fluorescence intensity of the cell was quantified using ImageJ software (version 1.42L; NIH, MD).

### 2.8 Cortical granules (CG) staining

CG staining was performed as previously described (Paola et al., 2015). Briefly, after MII oocytes incubation in CPP (10 µM), at 37°C, and different times (15, 30, 60 min), ZP was removed by brief incubation in acid Tyrode and oocytes were fixed in paraformaldehyde for 1 h at RT. Then, oocytes were washed in BS, and permeabilized with Triton X-100 for 15 min. After 3 washes in BS, cells were incubated in 25 μg/mL Rhodamine-labeled Lens Culinaris Agglutinin (LCA) in BS for 30 min, after which they were washed again in BS. Cells were mounted in Vectashield mounting medium (Vector Laboratories, Burlingame, CA) between slide and coverslip (flattened mounting), sealed, and stored at 4°C until viewing.

### 2.9 Recombinant permeable tetanus toxin light chain (CPP-TeTx) design and purification

The TeTx light chain purification was performed as described by Mayorga et al., 2019 (Mayorga et al., 2020). The plasmid DNA encoding His6-tetanus toxin light chain in pQE3-Qiagen (previously provided by T. Binz from Medizinische Hochschule Hannover, Hannover, Germany) was transformed into *E. coli* XL-1Blue (Stratagene, La Jolla, CA, United States), and protein expression was induced overnight at 20°C with 0.2 mM IPTG. Purification of recombinant His_6_-tetanus toxin (TeNT) was accomplished according to the QIAexpressionist (www.qiagen.com). The CPP peptide (RRRQRRKRRRRQ) and the recombinant toxin (His6-TeNT) were cross-linked by Trilinks Biotechnology under the manufacturing protocol. The primary amines of both toxin and peptide were linked to succinimidyl-4-formyl benzamide (S-4FB) and succinimidyl-6-hydrazino-nicotinamide (SHyNic), respectively. The modified proteins and peptides were then conjugated by a specific chemical reaction between HyNic and 4FB in a particular molar ratio (3:1 M equivalent) at RT by 3 h incubation with aniline 100 mM as a catalyst. After the conjugation reaction, the cross-linkers reagents surplus was removed and the CPP-TeTx was purified on a Sephadex G25 column (MP Biomedicals) in a medium suitable for oocyte incubation (MEM/HEPES). The purity was confirmed by SDS-PAGE analysis.

### 2.10 CPP-TeTx incubation and inmunofluorescence assay

Incubation of MII oocytes was performed in CPP-TeTx (4 μM) for 15, 30 and 60 min, at 37°C on a thermal stage. After that, ZP was removed by brief incubation in acid Tyrode and the oocytes were fixed in paraformaldehyde for 1 h at RT. Cells were then washed in BS, and permeabilized with Triton X-100 for 15 min. After 3 washes in BS, incubation was performed in Mouse Anti-6X His (Abcam) primary antibody (1:20) for 1 h at RT. After 3 washes in BS, oocytes were incubated for 1 h in DyLight 488 donkey anti-mouse secondary antibody (3 ng/μl, Jackson ImmunoReasearch). After 3 washes, cells were flat mounted in Vectashield mounting medium (Vector Laboratories, Burlingame, CA) on a slide, sealed, and stored at 4°C until visualization.

### 2.11 Parthenogenetic activation with strontium chloride (SrCl_2_)

After MII oocytes incubation in 10 μM FAM-CPP or 4 μM pTxTe for 60 min, at 37°C, the cortical reaction was induced by parthenogenetic activation with SrCl_2_ (30 mM) in calcium and magnesium free CZB (85.35 mM NaCl, 4.83 mM KCl, 1.18 mM KH_2_PO4, 110 μM EDTA.2Na, 12 mM NaHCO_3_ 25, 270 μM Na pyruvate, 52 mM Na lactate, supplemented with 0.001% Gentamicin, 0.01% PVA, 1 mM Glutamine), for 1 h at 37°C in a humidified CO_2_ (5%) atmosphere. Then, ZP was removed by brief incubation in acid Tyrode and oocytes were fixed in paraformaldehyde for 1 h at RT. Cells were washed in BS, and permeabilized with Triton X-100 for 15 min. After 3 washes in BS, cells were incubated in 25 μg/mL Rhodamine-labeled Lens Culinaris Agglutinin (LCA) in BS for 30 min, after which they were washed again in BS. Cells were flattened-mounted in Vectashield mounting medium (Vector Laboratories, Burlingame, CA) on a slide, sealed, and stored at 4°C. To demonstrate the specificity of the effect of pTxTe, a group of oocytes was incubated with N,N,N′,N′-Tetrakis (2-pyridylmethyl) ethylenediamine (TPEN), a zinc chelator permeable to cells with a high affinity for zinc. Oocytes were treated with 10 μM TPEN during 15 min at 37°C prior activation with SrCl_2_.

### 2.12 Microscopy imaging and quantification

The images were acquired with a confocal laser scanning microscope (FV1000, Olympus), using a PLAPON 60x/NA1.42 oil immersion objective lens, at a pixel resolution of ×512 512. The cell images were taken at flat optical fields of the equatorial zone (to analyze either the fluorescence of CPP or the immunolabeled CPP-TeTx) or at the cortical zone (to analyze cortical granule density). The confocal acquisition parameters were constant for all images captured. Using the computer-assisted image quantification software ImageJ (version 1.42L; NIH, MD), the absolute fluorescence intensity of oocytes treated with CPP or CPP-TeTx was quantified by drawing a circumference that took the cytoplasm of the cell, but not the cortical zone, and whose diameter was maintained for all the cells analyzed (Figure 1B). To quantify the density of CG through ImageJ, images were deconvoluted to obtain greater sharpness. The mean obtained from the counting of CG present in four non overlapping equal areas from the oocyte cortex, was used to determine CG density per 100 μm^2^ (CG/100 μm^2^) for each cell.

### 2.13 Data analysis

Experiments were repeated at least three times. The number of oocytes used for each experiment is indicated in the figure or in legends. Data analysis was performed with GraphPad Prism 8 software. Depending on data distribution (whether parametric or not), statistical significance was determined by Student/Mann Whitney *t*-test or One-way Analysis of Variance (ANOVA)/Brown-Forsythe and Welch ANOVA/Kruskal–Wallis tests followed by *post hoc* test for multiple comparisons. Data are expressed as mean ± SEM and *p* < 0.05 is considered statistically significant.

## 3 Results

### 3.1 The fluorescent cell permeable peptide K(5-FAM)R_9_C is able to cross both zona pellucida and plasma membrane of mouse oocytes

In this study, we investigated the internalization ability of cell-penetrating peptides across the zona pellucida and plasma membrane of mouse oocytes. As a representative CPP model, we selected KR_9_C, a peptide comprising nine consecutive arginine residues. Previous research has indicated that oligoarginine sequences consisting of eight to twelve consecutive residues demonstrate optimal internalization capability for small molecules in mammalian cells (Mitchell et al., 2000; Mueller et al., 2008; Tünnemann et al., 2008). To observe the intracellular behavior of the CPP using fluorescence microscopy, we introduced the 5-FAM (5-carboxyfluorescein) into the peptide sequence. Specifically, FAM was conjugated covalently to the lysine residue (see Materials and Methods), resulting in the peptide sequence K(5-FAM)R_9_C, which will be referred to as FAM-CPP hereinafter.

To evaluate if this FAM-CPP was able to cross the oocyte’s plasma membrane, we analyzed the fluorescence intensity in Metaphase II (MII or mature) oocytes after cells incubation at 37 °C in the presence (or absence) of 10 µM CPP. For these assays, MII oocytes with zona pellucida were used.

After fixation, cells were mounted in two different manners: in “chamber” or “flattened” between slide and coverslip (see schematic representation of mountings in Figure 1A). To detect exclusively the cytoplasmic fluorescence, only equatorial zone of the cells was imaged. Confocal microscopy images of oocyte’s equatorial zone showed that FAM-CPP accumulated on the outside of cells and in the polar body; thus, we only analyzed the fluorescence intensity of the equatorial region within the red circle as indicated in the scheme of Figure 1B. The analysis of fluorescence intensity showed that the flattened method was the best mounting manner to obtain a significant signal compared to control cells (Figure 1C). FAM-CPP showed a diffuse pattern of distribution in oocyte cytoplasm, while control cells incubated only with FAM (without CPP) did not shown fluorescence. These results indicated that the fluorescent CPP was able to cross the plasma membrane of mature oocytes with zona pellucida and that the flattened method was the best cell mounting to analyze the FAM-CPP internalization in the assayed conditions.

**FIGURE 2:**
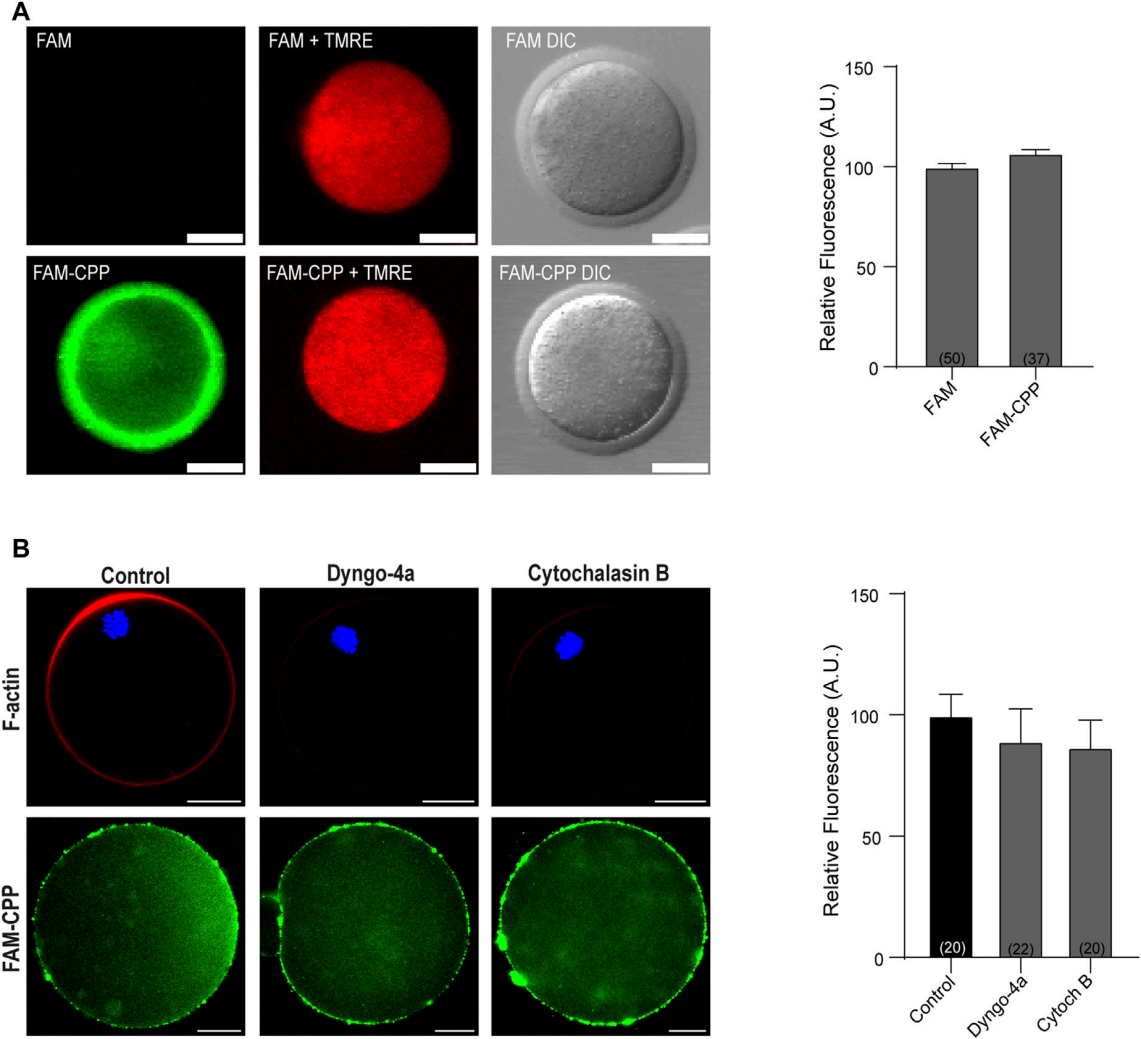
FAM-CPP does not affect cell viability and enters oocytes by translocation. (**A)** Effect of incubation with FAM-CPP on mitochondrial membrane potential (MMP). MII oocytes with ZP were incubated with 10 μM FAM or FAM-CPP during 1 h, and then incubated in the probe TMRE (red) to analyze MMP *in vivo*. Left: representative confocal microscopic images of oocytes. Scale bar: 5 μm. Right: relative fluorescence intensity at different treatments. The data represent mean ± SEM. Numbers in parentheses at the base of the bar represent the total number of oocytes. Comparisons between FAM and FAM-CPP were made by t Student test (*p* < 0.12). DIC: differential interference contrast. (**B**) Effect of endocytosis inhibition on FAM-CPP penetration. MII oocytes were preincubated with 80 μM Dyngo-4a or 20 μM Cytochalasin B (Cytoch B) prior the incubation with 10 μM FAM-CPP (green). Left: upper panel, representative confocal microscopic images of oocytes stained with Rhodamine-Phalloidin 555 (F-actin, red) and mounted in chamber; lower panel, representative confocal microscopic images of oocytes incubated in FAM-CPP after endocytosis inhibition. Right: relative fluorescence intensity at different treatments. The data represent mean ± SEM. Numbers in parentheses at the base of the bar represent the total number of oocytes. Comparisons between control, Dyngo and CB were made by Kruskal–Wallis and Dunn’s multiple comparisons test (*p* = 021).

One of the fundamental conditions for a CPP to be viable as a biotechnological tool is that it has low or no cytotoxicity. Although there are studies with cationic peptides (such as the polyarginine used in this work) that indicate that they have low toxicity and are tolerated by cells at high concentrations (Dinca et al., 2016), we first analyzed the viability of mature oocytes incubated with CPP by determining the mitochondrial membrane potential (MMP). As shown in Figure 2A, after 1 h incubation at 37°C in presence of 10 µM CPP, mature oocytes were alive, indicating that the FAM-CPP was not toxic for the cells. Then, to rule out that that FAM-CPP was entering by endocytosis, we preincubated oocytes with two different endocytosis inhibitors before CPP incubation: Dyngo-4a and Cytochalasin B. Dyngo-4a is a dynamin inhibitor that inhibit clathrin-mediated endocytosis and perturb the actin dynamic (Ferguson and De Camilli, 2012; McCluskey et al., 2013). Cytochalasin B is a known reagent that disassembly F-actin cytoskeleton (Cooper, 1987). Mouse oocytes were preincubated in presence of the endocytosis inhibitors prior FAM-CPP incubation. After fixation, oocytes were either flatten mounted to analyze the cytoplasmic FAM-CPP fluorescence or permeabilized to stain F-actin (see details in Materials and Methods) As shown in Figure 2B, both Dyngo-4a and Cytochalasin B disrupted cortical F-actin when compared to control condition. However, FAM-CPP was observed in the cytoplasm indicating that, even when endocytosis was inhibited, the CPP was internalized. These results suggest that the translocation of FAM-CPP take place by a direct penetration mechanism.

**FIGURE 3:**
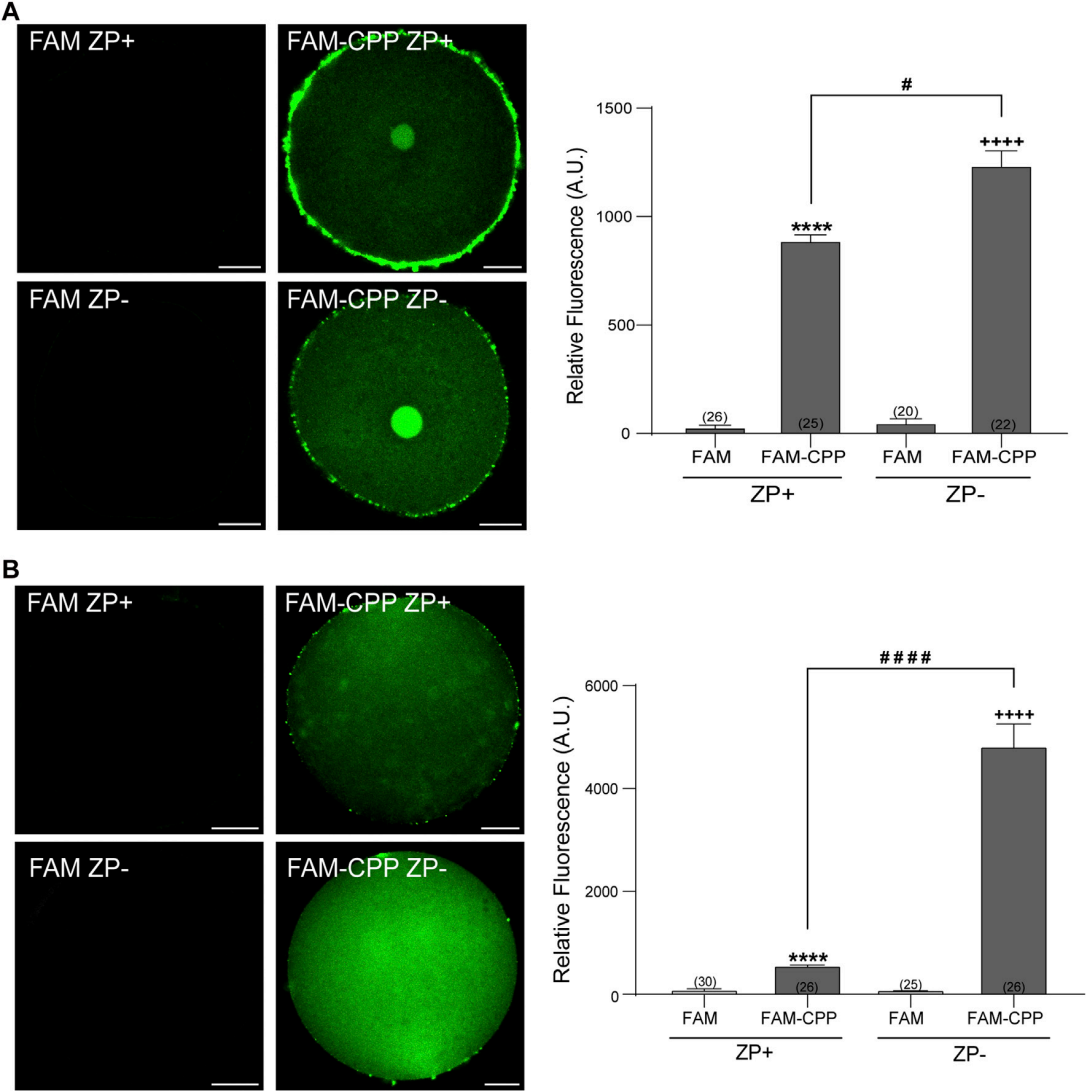
Effect of zona pellucida (ZP) on FAM-CPP internalization. GV oocytes **(A)** and MII oocytes **(B)** were incubated in FAM-CPP (4 and 10 μM, respectively) at 37°C, with (ZP+) or without (ZP-) zona pellucida. Left **(A,B)**. Representative confocal microscopy images taken at equatorial plane of the cell. Scale bar: 15 μm. Right **(A,B)**. Histogram showing relative fluorescence intensity for different treatments. The data represent mean ± SEM calculated from independent experiments. Numbers in parentheses at the base of the bar represent the total number of oocytes. Comparisons between FAM group and treatments were made by Kruskal–Wallis and Dunn’s multiple comparisons test (*p* < 0.0001) for GV oocytes, and Brown-Forsythe and Welch ANOVA tests, and Games-Howell’s multiple comparisons test (*p* < 0.0001) for MII oocytes. * (ZP+) and + (ZP-) represent statistically significant differences compared to control. # represent significant differences between groups.

The zona pellucida (ZP) is a glycoprotein matrix that functions as a protective barrier, filtering substances from the extracellular space (Gupta et al., 2012). Because this barrier must be crossed to introduce substances into the oocytes, we analyzed the effect of the presence/absence of ZP on the FAM-CPP internalization. For this assay, the ZP was removed before incubating with the fluorescent CPP as explained in Materials and Methods. As shown in Figure 3, both GV oocytes and MII eggs without ZP were able to incorporate significatively more FAM-CPP than cells with ZP, indicating that ZP hinders the fluorescent CPP incorporation. Nevertheless, for the following assays, we decided to use oocytes with ZP because they are easier to manipulate.

Taken together, these results showed that the FAM-CPP can cross both zona pellucida and plasma membrane of MII oocytes (with and without ZP) and does not affect cell viability. Nevertheless, it is worthy to mention that, even if the peptide can cross de ZP, the internalization is significatively reduced in oocytes with ZP.

### 3.2 Effect of concentration, time, and temperature on CPP internalization during mouse oocyte maturation

There is evidence in the literature on the differential influence of concentration, time and temperature on the internalization of CPP (Fretz et al., 2007; Madani et al., 2011; Copolovici et al., 2014). However, often the mentioned factors act in conjunction with other factors such as the nature of the CPP and the type of cell used. For this reason, we tested how those parameters -concentration, time, and temperature-participate in the internalization of FAM-CPP in two different stages of mouse oocyte maturation: Germinal Vesicle (GV, immature) and Metaphase II (MII, mature) oocytes.

**FIGURE 4:**
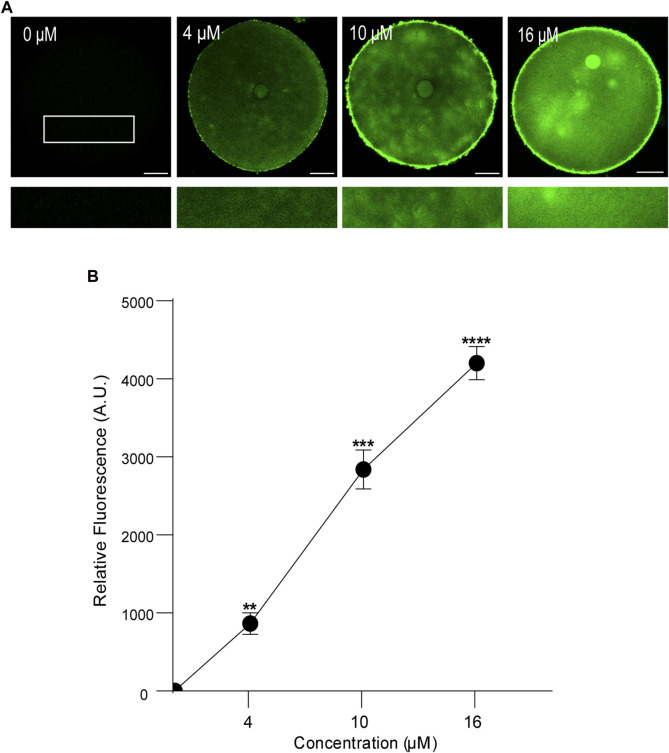
Effect of concentration on FAM-CPP internalization in GV mouse oocytes. Germinal Vesicle (GV) oocytes were incubated at different concentrations of FAM-CPP at 37 °C for 1 h. **(A)** Upper panels show representative confocal microscopy images taken at the equatorial plane of the cell inside the red circle (as represented in Figure 1B). Lower panels show magnification taken from rectangles of the same size represented only in 0 μM. Scale bar: 15 μm. **(B)** Relative fluorescence intensity at different FAM-CPP concentrations. The data represent mean ± SEM calculated from independent experiments. Comparisons with 0 μM were made by Kruskal–Wallis and Dunn’s multiple comparisons test (*p* < 0.0001). * represents significant differences. Number of cells analyzed: 0 μM: 26; 4 μM: 32; 10 μM: 26; 16 μM: 27.

**FIGURE 5:**
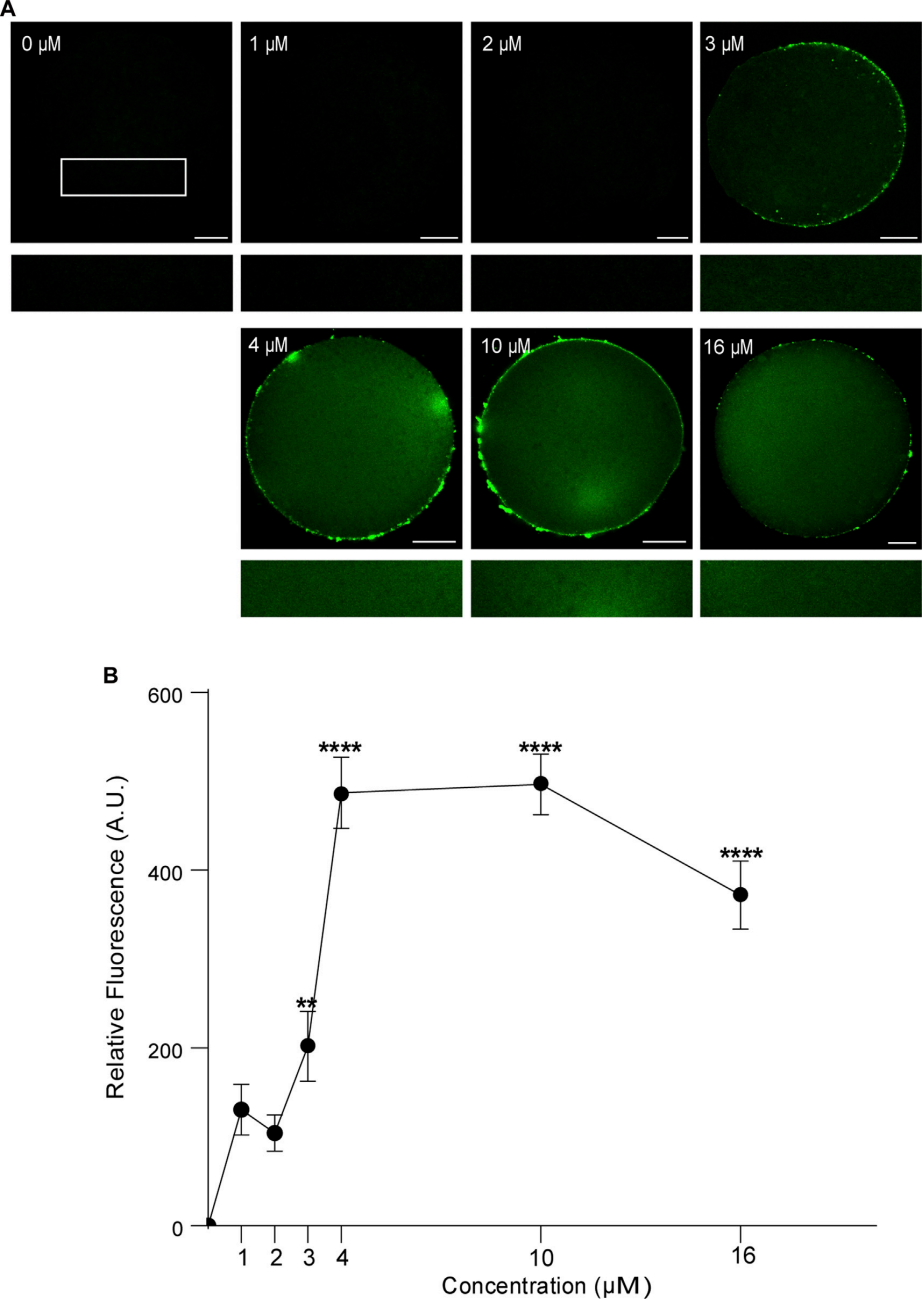
Effect of concentration on FAM-CPP internalization in MII mouse oocytes. Metaphase II (MII) oocytes were incubated at different concentrations of FAM-CPP at 37°C for 1 h. **(A)** Upper panels show representative confocal microscopy images taken at the equatorial plane of the cell inside the red circle (as represented in Figure 1B). Lower panels show magnification taken from rectangles of the same size represented only in 0 μM. Scale bar: 15 μm. **(B)** Relative fluorescence intensity at different FAM-CPP concentrations. The data represent mean ± SEM calculated from independent experiments. Comparisons with 0 μM were made by Kruskal–Wallis and Dunn’s multiple comparisons test (*p* < 0.0001). * represents significant differences. Number of cells analyzed: 0 μM: 41; 1 μM: 31; 2 μM: 27; 3 μM: 26; 4 μM: 45; 10 μM: 54; 16 μM: 50.

First, we analyzed the concentration effect on the FAM-CPP internalization in GV and MII mouse oocytes. Different concentrations (1–16 µM) were tested in immature and mature oocytes with ZP. Again, cells were imaged at equatorial region inside the red circle as explained previously. As shown in Figures 4A, B, the increasing of fluorescence intensity was directly proportional to FAM-CPP concentration in GV oocytes. CPP was homogeneously distributed on the entire cytoplasm and reached the nucleus (or Germinal Vesicle). In other words, FAM-CPP internalization was concentration-dependent in immature oocytes. When the concentration effect on the FAM-CPP internalization was analyzed in MII oocytes, the fluorescence intensity reached a statistically significant plateau between 4 μM and 10 µM and diminished significantly at 16 µM (Figures 5A, B). These results indicate that, similarly to observed in GV oocytes, FAM-CPP internalization was concentration-dependent in MII oocytes until 4 uM, however it reached a plateau at higher concentrations.

**FIGURE 6:**
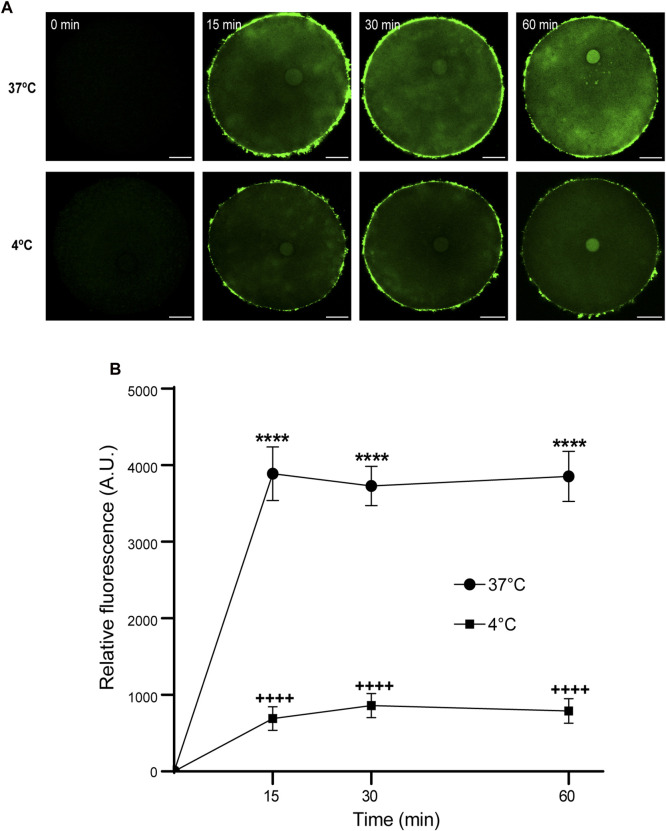
Effect of time and temperature on FAM-CPP internalization in GV oocytes. Oocytes were incubated with 10 μM FAM-CPP at 37 °C or 4°C, for 15, 30, and 60 min. **(A)** Representative confocal microscopy images taken at equatorial plane of the cell. Scale bar: 15 μm. **(B)** Relative fluorescence vs. time (min), at 4°C and 37°C, and relative to untreated group (0 min). The data represent mean ± SEM calculated from independent experiments. Comparisons between control and treatments were made by Kruskal–Wallis and Dunn’s multiple comparisons test (*p* < 0.0001). * (37°C) and + (4°C) represent significant differences compared to 0 min. Number of cells analyzed: 4°C: 0 min: 27; 15 min: 30; 30 min: 29; 60 min: 24; 37°C: 0 min: 26; 15 min: 41; 30 min: 60; 60 min: 52.

**FIGURE 7:**
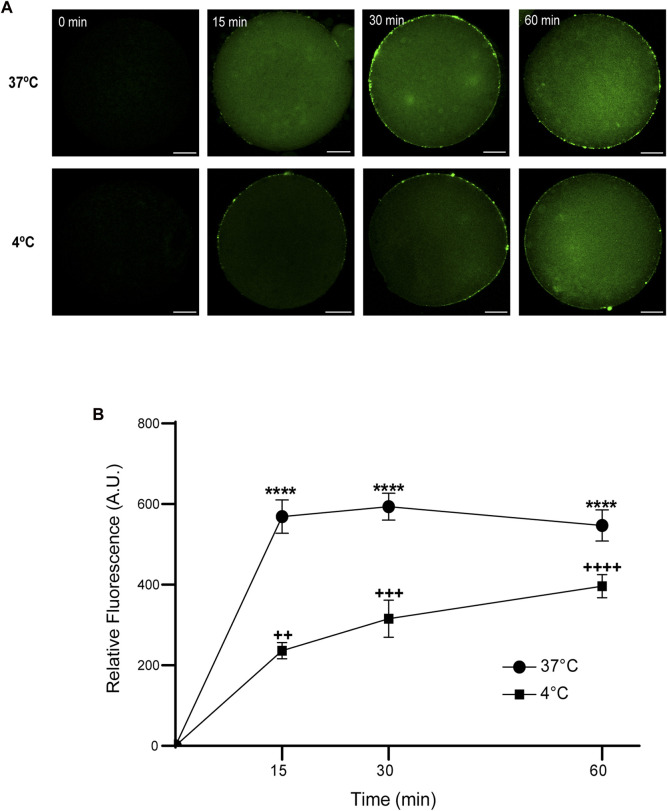
Effect of time and temperature on the internalization of FAM-CPP in MII oocytes. Oocytes were treated with 10 μM FAM-CPP at 37°C or at 4°C, for 15, 30 and 60 min. **(A)** Representative confocal microscopy images taken at equatorial plane of the cell. Scale bar: 15 μm. **(B)** Relative fluorescence vs. time (min), at 4°C and 37°C, and relative to untreated group (0 min). The data represent mean ± SEM calculated from independent experiments. Comparisons between control and treatments were made by Kruskal–Wallis and Dunn’s multiple comparisons test (*p* < 0.0001). * (37°C) and + (4°C), represent significant differences compared to control. Number of cells analyzed: 4°C: 0 min: 47; 15 min: 38; 30 min: 45; 60 min: 59; 37°C: 0 min: 50; 15 min: 48; 30 min: 44; 60 min: 43.

Then, we analyzed the effect of time and temperature simultaneously on FAM-CPP internalization for each maturation stages. GV oocytes were collected and incubated in CZB medium with 10 µM FAM-CPP for 15, 30, and 60 min at 4°C or 37°C. As shown in Figure 6, the FAM-CPP internalization occurred at both temperatures and reached a statistically significant plateau at 15 min. Nevertheless, the FAM-CPP internalization at 4°C was statistically lower than 37°C (Figures 6A, B). Similar assays were performed using MII oocytes. Cells were collected and incubated in CZB medium with 10 µM FAM-CPP for 15, 30, and 60 min at 4°C or 37°C. As shown in Figure 7, similar results were obtained when MII oocytes were incubated at 37°C: the intensity of CPP fluorescence reached a plateau at 15 min. Nevertheless, when the FAM-CPP incubation took place at 4°C, the FAM-CPP internalization was dependent on the incubation time (Figures 7A, B).

Altogether, these results demonstrate that the FAM-CPP internalization in mouse oocytes depends on concentration, time, temperature, and the oocyte maturation stages.

### 3.3 The internalization of FAM-CPP does not affect the density of cortical granules in mature oocytes

**FIGURE 8:**
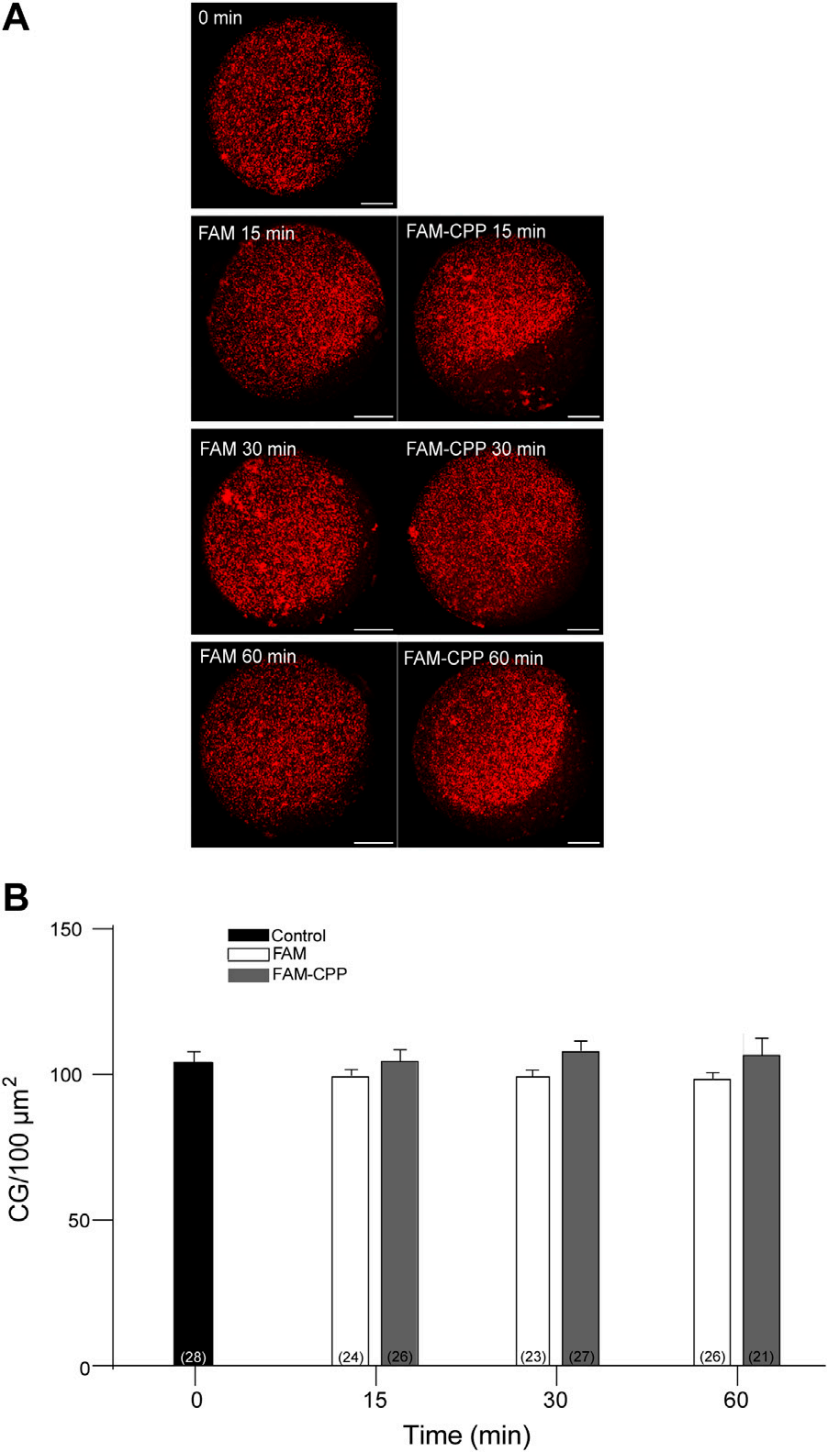
FAM-CPP internalization does not perturb cortical granules density in MII oocytes. Oocytes were incubated with 10 μM FAM-CPP and FAM (control) for 15, 30 and 60 min at 37 °C. After fixation, cortical granules (CG) were stained with Rhodamine-LCA (red). **(A)** Representative confocal microscopic images of oocytes taken at cortical plane of the cell. Scale bar: 15 μm. **(B)** Histogram showing CG density for different times and relative to untreated group (0 min) set as 100%. The data represent mean ± SEM calculated from at least three independent experiments. Comparisons between control and treatments were made by Ordinary one-way ANOVA and Tukey’s multiple comparisons test (*p* > 0.05). Numbers in parentheses at the base of the bar represent total number of oocytes.

Cortical granules (CG) are organelles localized in the cortical region of mature mouse oocytes and are involved in the cortical reaction. This process is an exocytotic event in which CG fuse with the oocyte plasma membrane after the fusion of the first spermatozoa. Thus, the secretion of CG content avoids the fusion of a second male gamete ensuring the embryo development. Considering that this work aimed to analyze the use of CPP to study cortical reaction in mature mouse oocytes, the next step was to investigate if the FAM-CPP internalization does perturb cortical granules density. Then, cortical granules density was evaluated for different incubation times in mature oocytes. For control and experimental conditions, cells were incubated with FAM and FAM-CPP, respectively. The results showed that, compared to control, the FAM-CPP incubation did not alter the cortical granule density at any assayed time in mature oocytes (Figure 8).

### 3.4 The permeable tetanus toxin is internalized into mature oocytes and inhibits cortical reaction

Cortical reaction is a calcium regulated exocytosis, in which cortical granules fuse with the oocyte plasma membrane after mouse oocyte activation. This fusion is mediated by SNARE proteins, and we have proposed a working model for cortical granules exocytosis in mouse oocytes (Paola et al., 2021). The SNARE proteins are proteolyzed by the light chains of the clostridial neurotoxins–tetanus toxin and botulinum toxin (Humeau et al., 2000; Schiavo et al., 2000). Tetanus toxin has two polypeptide chains: the heavy and the light chain. The heavy chain mediates binding, internalization, and translocation of the light chain to the cytosol, and the light chain inhibits exocytosis by cleaving VAMP1, VAMP2 or VAMP3 (Dong et al., 2019). In addition, the catalytic activity of the light chain is zinc-dependent and is used as a tool for the study of exocytosis in different secretory cells (Ahnert-Hilger et al., 1989; Bittner et al., 1989). Previously we have demonstrated that VAMP1 and VAMP3 are expressed in mouse oocytes (Paola et al., 2021). Considering that FAM-CPP internalization is innocuous for mouse oocytes (Figure 2A) and does not perturb cortical granules localization (Figure 8), we hypothesized that a permeable tetanus Toxin light chain (CPP-TeTx) might inhibit cortical reaction by cleaving the VAMPs isoforms expressed in mouse oocytes.

**FIGURE 9:**
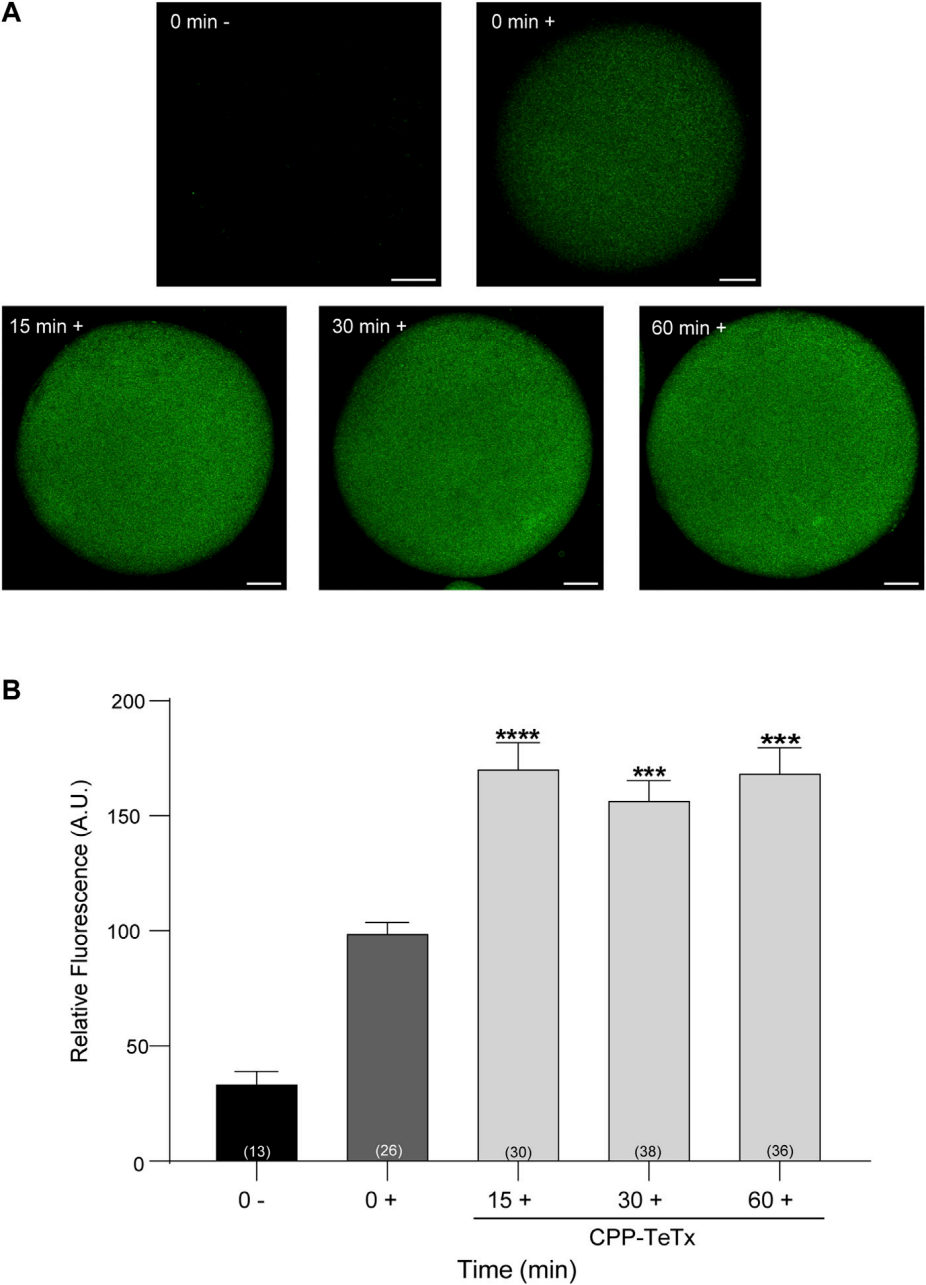
Permeable tetanus toxin (CPP-TeTx) is internalized into MII oocytes. Oocytes were incubated with 4 μM CPP-TeTx for 1 h at 37°C. The permeable TeTx was visualized by immunofluorescence assay. **(A)** Representative confocal microscopic images of oocytes taken at equatorial plane of the cell. Scale bar: 15 μm. **(B)** Histogram showing relative fluorescence intensity for different times and relative to 0+ group set as 100%. The data represent mean ± SEM calculated from independent experiments. Comparisons between control and treatments were made by Kruskal–Wallis and Dunn’s multiple comparisons test (*p* < 0.0001). * represents significant differences between control (0+) and treatments. Numbers in parentheses at the base of the bar represent total number of oocytes. Minus sign (−): fixed oocytes were treated only with secondary antibody. Plus sign (+): fixed oocytes were treated with primary and secondary antibodies.

First, we purified the CPP-TeTx as a His6-tagged protein as described in Materials and Methods. We tested if this permeable toxin could internalize into mouse mature oocytes. Because CPP-TeTx is not fluorescent, we verified the protein internalization by immunofluorescence assays using a His6 antibody. As shown in Figure 9, CPP-TeTx was detected into mature oocytes incubated by 15, 30 and 60 min at 37°C (Figure 9A). In this case, two controls were performed: one, to determine the unspecific signal from the secondary antibody (0 min-, incubated only with secondary antibody), and the other, to determine the unspecific signal from histidines present in mature oocytes (0 min+, incubated with primary and secondary antibody). Figure 9B shows that the fluorescence intensity for all analyzed times was statistically higher than 0 min + control and time independent. These results are similar to that obtained with FAM-CPP (see Figure 7) and indicate that CPP-TeTx was internalized into mouse mature oocytes.

**FIGURE 10:**
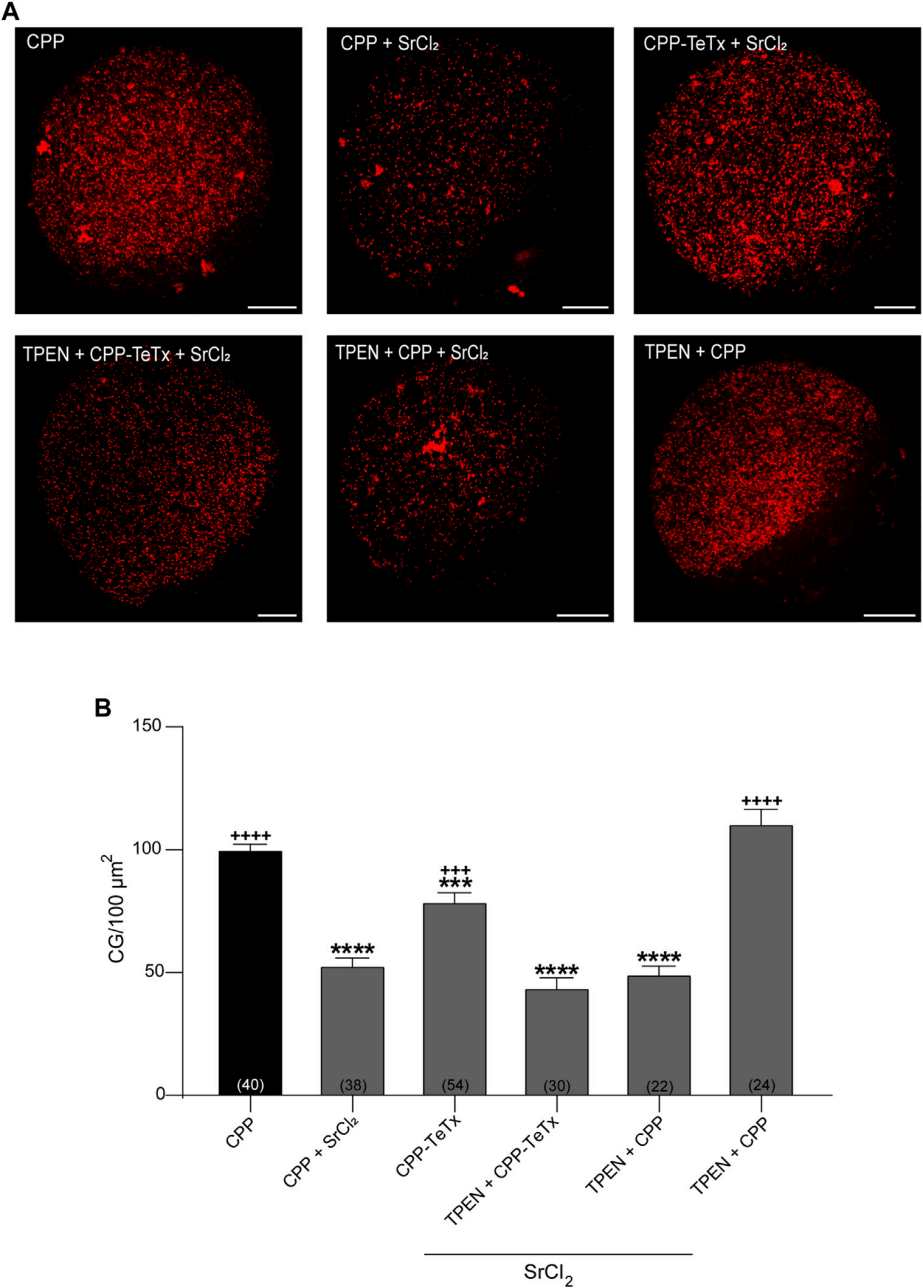
Permeable tetanus toxin (CPP-TeTx) inhibits cortical granules exocytosis in MII oocytes. Mature oocytes were incubated in 4 μM CPP-TeTx for 1 h at 37 °C before parthenogenetic activation with SrCl_2_ (see text for details). **(A)** After fixation, cortical granules (GC) were stained with Rhodamine-LCA (red); representative confocal microscopic images of oocytes taken at cortical plane of the cell. Scale bar: 15 μm. **(B)** Histogram showing CG density for different treatments and relative to CPP (control) set as 100%. The data represent mean ± SEM calculated from independent experiments. Comparisons between CPP and different conditions were made by Brown-Forsythe and Welch ANOVA tests, and Games-Howell’s multiple comparisons test (*p* < 0.0001). * represents significant differences compared to CPP; + represents significant differences compared to CPP + SrCl_2_. Numbers in parentheses at the base of the bar represent total number of oocytes.

Second, to test the functionality of CPP-TeTx during parthenogenetically activated cortical reaction, we analyzed the function of CPP-TeTx using our functional assay. This functional assay consists of quantifying and comparing the cortical granules density in control oocytes (without activator) and in strontium-activated oocytes (positive control, with parthenogenetic activator). The difference between these two conditions is a functional measurement of cortical granule exocytosis (Supplementary Figure S1). Considering that the sequence of permeable peptide is similar for both FAM-CPP and CPP-TeTx, we tested if FAM-CPP internalization may affect the cortical reaction. As shown in Supplementary Figure S1, FAM-CPP did not perturb the cortical reaction activated by strontium chloride. Then, if prior strontium activation an inhibitor is present (CPP-TeTx), cortical granules exocytosis will be inhibited, and cortical granules density will be significantly higher than positive control. Mature oocytes with ZP were incubated in the presence of 4 µM CPP-TeTx at 37 °C during 1 h. Similarly to FAM-CPP (see Figure 5), 4 uM CPP-TeTx was the minimum concentration to reach the internalization plateau. Then, cortical reaction was activated by strontium chloride. Before fixation, ZP of the treated oocytes was removed and cortical granules were stained with Rhodamine-Lens Culinaris Agglutinin (LCA) to evaluate cortical granules density as a measure of cortical reaction (see Materials and Methods for details). As shown in Figure 10, CPP-TeTx significantly inhibited the cortical reaction activated by SrCl_2_. CPP-TeTx inhibited significantly cortical reaction by about 50%, suggesting that other tetanus toxin-insensitive protein might be involved in this secretory process. Next, considering that the catalytic activity of the light chain of tetanus Toxin is zinc-dependent, we analyzed the effect of zinc chelation to demonstrate the specificity of CPP-TeTx. We used N,N,N′,N′- Tetrakis(2-pyridylmethyl) ethylenediamine (TPEN), a cell-permeable zinc chelator with a high affinity for zinc. Incubation of CPP-TeTx treated oocytes in 10 μM TPEN prevented the inhibition of cortical granule exocytosis, confirming the specificity of the effect of CPP-TeTx (Figure 10). TPEN incubation alone did not alter the cortical reaction in presence or absence of SrCl_2_ (Figure 10).

Altogether, these results confirmed our hypothesis and demonstrated that the permeable tetanus toxin inhibits cortical granule exocytosis in mouse oocytes.

## 4 Discussion

In this work, we aimed to assess the potential of cell-penetrating peptides (CPP) as a non-invasive approach for studying cortical reaction in mouse oocytes. We and other groups have characterized the molecular mechanism of this secretory process by microinjecting different proteins and antibodies into oocyte cytoplasm (Paola et al., 2015; Bello et al., 2016; Wang et al., 2016; Mehlmann et al., 2019; Zhu et al., 2020; Paola et al., 2021). The intracytoplasmic microinjection is an invasive method that requires specialized microinjection equipment and skilled personnel. Nevertheless, it is the only available method to cross both structures the zona pellucida (ZP) and the oocyte plasma membrane. ZP is a unique structure of female gamete, and is formed by glycoproteins that functions as a protective barrier, filtering substances from the extracellular space (Gupta et al., 2012). CPPs possess the ability to cross cell membranes naturally (Koren et al., 2012); however, the CPP ability to cross ZP has been poorly explored. Previous studies have demonstrated that hydrophilic arginine-rich cell-penetrating peptides have optimal internalization capability for small molecules in mammalian cells (Mitchell et al., 2000; Mueller et al., 2008; Tünnemann et al., 2008). Presently, there is a limited amount of literature available on the utilization of CPPs in oocytes (Kwon et al., 2009; Kwon et al., 2013; Yang et al., 2014; Jeon et al., 2019; Zhu et al., 2021), and none of these studies extensively examine the internalization of CPPs into mouse oocytes.

First, we evaluated the ability of CPPs to cross both the zona pellucida and plasma membrane of immature (GV) and mature (MII) mouse oocytes within a 1 h incubation period. To this end, we specifically used K(5-FAM)R_9_C, a fluorescent peptide rich in arginine residues conjugated with FAM (FAM-CPP). Our results indicate that FAM-CPP successfully enters the oocytes (GV and MII), displaying a diffuse pattern of cytoplasmic distribution (Figure 1). Two primary mechanisms have been proposed for CPP internalization: endocytosis and direct penetration (Gestin et al., 2017). Direct penetration of fluorescent CPPs into living cells is expected to result in a uniform and diffuse labeling pattern, while endocytosis leads to a granular or punctuate pattern due to the labeled CPP becoming trapped in vesicular organelles (Pooga and Langel, 2015). Therefore, based on our observations that FAM-CPP was internalized when endocytosis was inhibited, we propose that FAM-CPP is internalized by a direct penetration mechanism, which does not affect cell viability.

We also examined the effect of peptide concentration on FAM-CPP internalization in GV and MII oocytes. It is noteworthy that a significantly higher amount of CPP enters GV oocytes compared to MII oocytes. This discrepancy can be attributed to the distinct stages of maturation at which these oocytes were found. Each stage exhibits unique characteristics, among which the structure of the ZP and the plasma membrane potential (Vm) could explain the difference in CPP internalization between GV and MII oocytes. In effect, Novo and others have reported that in GV oocytes, the porosity of the ZP is 2.5 times higher than that of mature oocytes (Novo et al., 2012). Regarding to Vm, electrophysiological investigations have shown that cell membrane depolarization occurs as *in vitro* meiotic maturation progresses from the GV stage (−46 mV) to the MII stage (−17 mV) in mouse oocytes. Moreover, hyperpolarization has been reported to increase CPP internalization, whereas depolarization impedes it. In silico modeling supports the idea that membrane hyperpolarization facilitates the formation of transient water pores, thereby enabling CPP translocation into cells (Trofimenko et al., 2021). Altogether, the increased CPP internalization in GV oocytes could be explained by a higher ZP porosity and by the hyperpolarization of the plasma membrane.

Additionally, we explored the influence of time and temperature on FAM-CPP internalization. Our analysis revealed rapid internalization of fluorescent CPP by GV oocytes at both 37°C and 4 °C. Although internalization was significantly higher at 37°C, fluorescence intensity reached a plateau within 15 min at both temperatures (Figure 6). Similarly, in MII oocytes, internalization of FAM-CPP reached a plateau within 15 min of incubation at 37°C. However, internalization was directly proportional to incubation time when mature oocytes were incubated at 4°C (Figure 7). To the best of our knowledge, there are no comparative studies on CPP internalization between immature and mature oocytes. Furthermore, investigations on CPP use in mouse oocytes are scarce. Jeon and collaborators have showed that exogenous translationally controlled tumor protein (TCTP conjugated with mCherry) was able to translocate into mature oocytes across the ZP after 4 h of incubation; however, internalization of the fluorescent TCTP in immature oocytes was not assayed (Jeon et al., 2019). Kwon and others showed that MPG, -a designed CPP comprised of the first 17 amino acids of N-terminus derived from glycine-rich region of the viral gp41 and the hydrophilic C-terminus from nuclear localization signal of the SV40 large T antigen-, was internalized in immature ZP-free oocytes after 1 h incubation period (S.-J. Kwon et al., 2009). Interestingly, MPG-EGFP showed a diffuse distribution pattern in GV oocytes; nevertheless, the authors only highlighted that the fluorescent MPG was limited to the cell periphery probably because of the presence of cortical granules (S.-J. Kwon et al., 2009). The same group also tested a 12-mer CPP from the conserved region of the human papillomavirus L1 capsid protein, LDP12. Nevertheless, CPP was not internalized in mature oocytes (with or without ZP) and was not assayed in immature oocytes (S. Kwon et al., 2013). In this work, we have demonstrated that CPP internalization observation depends on the mounting method. In fact, we showed that the best mounting method for obtaining a significant signal was the flattened method (Figure 1).

The final aim of analyzing CPP permeation into mouse oocytes was to utilize CPP as a non-invasive tool for studying cortical reaction. Cortical reaction is a calcium regulated exocytosis, in which cortical granules fuse with the oocyte plasma membrane after mouse oocyte activation. This fusion process is mediated by SNARE proteins, and we have proposed a working model for cortical granules exocytosis in mouse oocytes (Paola et al., 2021). Previously, we demonstrated that VAMP1 and VAMP3 -two SNARE sensitive to tetanus toxin-are expressed in mouse oocytes (Paola et al., 2021). Tetanus toxin has two polypeptide chains: the heavy and the light chain. The heavy chain mediates binding, internalization, and translocation of the light chain to the cytosol, whereas the light chain inhibits exocytosis by cleaving VAMP1, VAMP2 or VAMP3 at specific and single sites (Dong et al., 2019). Consequently, after confirming that FAM-CPP internalization did not perturb cortical granules distribution (Figure 8), we proceeded to investigate whether permeable TeTx could inhibit cortical granule exocytosis during the parthenogenetically activated cortical reaction in mouse oocytes. CPP-TeTx effectively was able to cross the zona pellucida and the plasma membranes of oocytes (Figure 9), and inhibited the cortical reaction activated by strontium in mature oocytes (see Figure 10). Therefore, these findings indicate that CPP-TeTx demonstrated to be functional in the cortical granule exocytosis assay, exhibiting a similar behavior to TeTx administered via microinjection (Paola et al., 2021).

In this work, we have characterized for the first time the internalization of a fluorescent arginine-rich cell-penetrating peptide in immature and mature oocytes. We demonstrated that the internalization of this cell-penetrating peptide (CPP) depends on different factors such as presence or absence of ZP, CPP concentration, temperature, incubation time, and oocyte maturation stage. We also bound this CPP to the light chain of tetanus toxin to demonstrate that this permeable version of tetanus toxin can cross ZP and plasma membrane and conserves its function. In fact, the permeable light chain of tetanus toxin inhibited the cortical reaction activated parthenogenetically by strontium chloride in mature oocytes.

Our findings indicate that arginine-rich cell-penetrating peptides can be utilized as a substitute method to intracytoplasmic microinjection for intracellular delivery of macromolecules into oocytes to study cortical granules exocytosis. This approach has several advantages over the traditional microinjection technique, including ease of use, efficiency, and versatility. The use of permeable peptides not only provides a new avenue for studying oocyte physiology but also offers potential applications in developing new therapies and a better understanding of the molecular mechanisms that regulate oocyte *in vitro* maturation, fertilization, and early embryonic development.

## Data Availability

The original contributions presented in the study are included in the article/Supplementary Material, further inquiries can be directed to the corresponding authors.
