# Supplementary figures and images for: The light chain of tetanus toxin bound to arginine-rich cell-penetrating peptide inhibits cortical reaction in mouse oocytes
Source: Front Cell Dev Biol. 2023 Nov 15;11:1259421. doi: 10.3389/fcell.2023.1259421 (PMC10684777; doi:10.3389/fcell.2023.1259421)

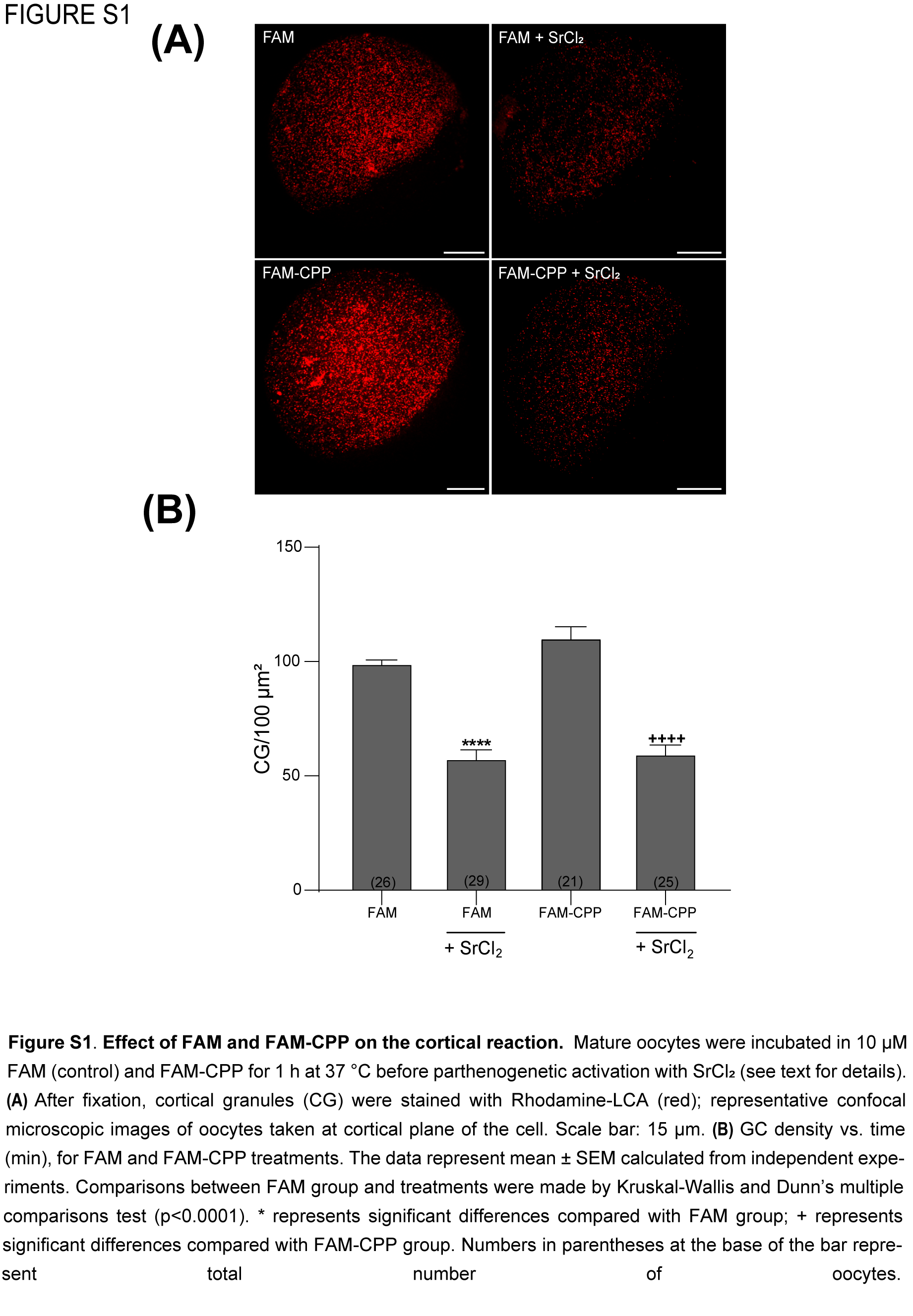

Supplement: Supplementary file 1 [file Image1.tif]
